# Activin A-Mediated Regulation of XT-I in Human Skin Fibroblasts

**DOI:** 10.3390/biom10040609

**Published:** 2020-04-14

**Authors:** Thanh-Diep Ly, Ricarda Plümers, Bastian Fischer, Vanessa Schmidt, Doris Hendig, Joachim Kuhn, Cornelius Knabbe, Isabel Faust

**Affiliations:** Institut für Laboratoriums- und Transfusionsmedizin, Herz- und Diabeteszentrum NRW, Universitätsklinik der Ruhr-Universität Bochum, Georgstraße 11, 32545 Bad Oeynhausen, Germany

**Keywords:** activin A, fibrosis, MAPK, proteoglycan, Smad, systemic sclerosis, xylosyltransferase

## Abstract

Fibrosis is a fundamental feature of systemic sclerosis (SSc) and is characterized by excessive accumulation of extracellular matrix components like proteoglycans (PG) or collagens in skin and internal organs. Serum analysis from SSc patients showed an increase in the enzyme activity of xylosyltransferase (XT), the initial enzyme in PG biosynthesis. There are two distinct XT isoforms—XT-I and XT-II—in humans, but until now only XT-I is associated with fibrotic remodelling for an unknown reason. The aim of this study was to identify new XT mediators and clarify the underlying mechanisms, in view of developing putative therapeutic anti-fibrotic interventions in the future. Therefore, we used different cytokines and growth factors, small molecule inhibitors as well as small interfering RNAs, and assessed the cellular XT activity and *XYLT1* expression in primary human dermal fibroblasts by radiochemical activity assays and qRT-PCR. We identified a new function of activin A as a regulator of *XYLT1* mRNA expression and XT activity. While the activin A-induced XT-I increase was found to be mediated by activin A receptor type 1B, MAPK and Smad pathways, the activin A treatment did not alter the *XYLT2* expression. Furthermore, we observed a reciprocal regulation of *XYLT1* and *XYLT2* transcription after inhibition of the activin A pathway components. These results improve the understanding of the differential expression regulation of *XYLT* isoforms under pathological fibroproliferative conditions.

## 1. Introduction

The skin is the largest organ in the human body and provides a protective barrier against microorganisms, injuries and water loss [1]. Approximately 70% of its dry weight is formed by the extracellular matrix (ECM) [2]. The synthesis, deposition and remodelling of the ECM is critical for the maintenance of tissue homoeostasis under physiological conditions. Despite being strongly regulated, disturbances of these processes can result in severe pathological diseases, such as hypertrophic scarring, scleroderma and fibrosis induced by surgery, radiotherapy or medication [3].

The hallmark of these fibroproliferative conditions is the activation and differentiation of fibroblasts into myofibroblasts due to physical or inflammatory insults. These cells secrete vast amounts of ECM proteins that accumulate in the tissue and impair proper organ function [4]. Furthermore, myofibroblasts are engaged in paracrine and autocrine interactions with their surrounding environment by secretion of growth factors and cytokines like activin A and transforming growth factor *β*1 (TGF*β*1), which are increased in many tissues during fibrosis [5,6,7].

Though activin A and TGF*β*1 belong to the TGF*β* superfamily and provide structural similarities, activin A is secreted as an active protein, whereas TGF*β*1 is secreted as an inactive precursor and requires activation [8,9]. Activin A plays a crucial role in wound healing and inflammation, therefore providing a critical link between the process of inflammation and fibrotic response in systemic sclerosis (SSc), the prototype of fibrotic skin diseases [10,11,12]. Activin A production is stimulated by several pro-fibrotic mediators, including TGF*β*1, interleukins (IL), endothelin-I, angiotensin-II and thrombin [6,13,14]. To promote fibroblast differentiation into myofibroblasts, exogenous activin A signals through combined type I and type II transmembrane kinase receptors, known as activin A receptor type 1B (ACVR1B) or activin receptor-like kinase 4 (ALK4) and ACVR2A or ACVR2B, which are shared by other TGF*β* ligands, such as myostatin, nodal and growth and differentiation factor 11 [12,15]. In all instances, ligand complex formation leads to phosphorylation and activation of intrinsic receptor kinase domains, which facilitate phosphorylation cascades. Similar to pro-fibrotic core mediator TGF*β*1, activin A mediates cellular responses via transcription factors mothers against decapentaplegic homolog 2/3 (Smad2/3)-dependent and non-canonical, Smad-independent signalling pathways, which involve the phosphorylation of the mitogen-activated protein kinases (MAPK) p38 MAPK, extracellular signal-regulated kinase (ERK) or c-Jun N-terminal kinase (JNK) in a cell type and cytokine-specific manner [16,17,18]. The activation of both the canonical as well as non-canonical signalling pathway has been demonstrated to be involved in fibrogenic changes [13,16,19]. 

Like TGF*β*1, activin A has a high affinity for the ECM by binding to heparin-sulphated proteoglycans (PG), such as perlecan, which are highly up-regulated in many human fibrotic conditions, including SSc [10,16,20]. The biological roles of PGs are related to their negatively charged glycosaminoglycan (GAG) chains that were post-translationally attached to the PG core proteins [20]. The formation of these GAG chains is initiated by xylosylation of specific serine units on preformed PG core proteins by the rate-limiting enzymes xylosyltransferase-I (XT-I) and -II (XT-II) (EC 2.4.2.26) [21]. 

The human XT-I and XT-II are type II transmembrane proteins, which are encoded by the genes *XYLT1* and *XYLT2*. Besides their similarities regarding enzyme function, their genes are differentially expressed in tissues [22,23]. Despite the intracellular localisation of the XT-I, the enzyme is shed from the membrane of the Golgi apparatus by an unknown mechanism that involves a cysteine protease [24]. The XT activity can be measured in the human serum and provides a reliable indicator for the present rate of PG biosynthesis and is a useful serum biomarker for the assessment of fibrotic processes in patients with SSc [25]. Additional studies using human primary fibroblasts as key players of ECM remodelling and tissue repair, have shown that *XYLT1* mRNA expression und extracellular XT activity correlates with the content of differentiated myofibroblasts measured by former myofibroblast marker α-smooth muscle actin (α-SMA) or expression of *ACTA2.* Furthermore, TGF*β*1 treatment of this cells leads to an increase in *XYLT1* expression and XT activity while *XYLT2* expression is unchanged after pro-fibrotic stimuli [26]. Therefore, XT-I expression is associated with persisting fibrosis and can be used as a myofibroblast differentiation marker [26,27]. However, little is known regarding the differential expressional regulation of both XT isoforms. Promoter analysis of XT-I revealed several transcription factor-binding sites for specificity protein 1 (SP1), early growth response factors (EGR) and Krueppel-like transcription factors (KLF), while the *XYLT2* promoter provides binding sites for SP1 family members. Interestingly, sequence analysis of the genomic DNA of 100 healthy blood donors revealed one single nucleotide variant (SNV), c.-1088C>A, in the *XYLT1* promoter region with an allele frequency of 38% that significantly reduced basal promoter activity by generation of a potential Smad3 binding site, but did not contribute to the measured serum XT activity of the donors [28,29,30]. 

Giving the missing link between *XYLT1* promoter analysis, identified transcription factors and the upstream cellular signalling mediating XT-I expression under fibrotic conditions, primary fibroblast cells provide a suitable model to study the relationship between exogenous pro-fibrotic stimuli and the underlying signal transduction pathways, which mediate the XT-I expression in PG biosynthesis and dermal myofibroblast differentiation.

## 2. Materials and Methods

### 2.1. Cell Culture

Adult normal human dermal fibroblasts (NHDF) from 37- to 50-year-old women and 45- to 57-year-old men were obtained from Genlantis (San Diego, CA, USA), Cambrex (Walkerville, MI, USA) and Coriell (Camden, NJ, USA). NHDF were maintained as monolayer cultures in 100 mm × 20 mm cell culture dishes with Dulbecco’s modified Eagle’s medium (DMEM; Thermo Fisher Scientific, San Diego, CA, USA) supplemented with 10% (v/v) foetal calf serum (Biowest, Nuaillé, France), 4 mmol/L l-glutamine and 100,000 *U*/L penicillin, 100 mg/L streptomycin, 0.25 mg/L amphotericin B (PAN Biotech, Aidenbach, Germany) at 37 °C, 5% CO_2_. Cells were used in Passage 4–9, corresponding to a cumulative population doubling levels of 7 to 17. Therefore, primary NHDF were subcultured with an expansion ratio not exceeding 1:3 upon near confluence using 0.05% trypsin (PAN Biotech, Aidenbach, Germany).

### 2.2. Cytokine and Inhibitor Treatment

To investigate the effect of the cytokines and inhibitors, NHDF were cultured for 24 h before treatment using a formerly established cell culture model [26]. In brief, cells were cultivated with a low density of 50 cells per mm^2^ on a hard tissue culture substrate (100 mm × 20 mm dish), promoting their trans-differentiation into proto-myofibroblasts. To avoid the effect of serum and to synchronize the cells, after an incubation time of 24 h fibroblasts were washed with Dulbecco’s phosphate-buffered saline (Thermo Fisher, San Diego, CA, USA) and maintained in serum-free DMEM unless otherwise stated. After 24 h, serum-starved cells were treated with 1 μg/L, 10 μg/L or 50 μg/L human recombinant activin A (HumanZyme Inc., Chicago, IL, USA), or 5 μg/L or 10 μg/L human recombinant TGF*β*1 for 3, 6, 24, 48 or 120 h. For inhibition studies final inhibitor concentrations of 10 μmol/L or 50 μmol/L SB431542 (Miltenyi Biotec GmbH, Bergisch Gladbach, Germany), 25 μmol/L SP600125, 10 μmol/L UO126, 20 μmol/L SB203580 (Cell Signaling Technology Inc., Danvers, MA, USA) or 10 μmol/L SIS3 (Merck KGaA, Darmstadt, Germany) diluted in serum-free DMEM were used. All inhibitors were dissolved in dimethyl sulfoxide or water and used according to the manufacturer’s instructions. Negative controls treated with solvent or vehicle were included for every sampling time. All experiments were performed in biological triplicates per number (*n*) of donor-derived primary cultures.

### 2.3. Nucleic Acid Extraction and Synthesis of Complementary DNA

Total RNA was extracted from cells using the RNA Spin Blood kit (Machery-Nagel, Düren, Germany) according to the manufacturer’s instructions. DNA extraction was performed using the DNA Spin Blood kit (Machery-Nagel, Düren, Germany). The NanoDrop 2000 spectrophotometer (Peqlab Erlangen, Germany) was used for measuring nucleic acid concentrations. An amount of 1 μg of total RNA was reverse transcribed into complementary DNA (cDNA) utilizing the SuperScript II Reverse Transcriptase kit (Thermo Fisher, Waltham, MA, USA) and diluted to 1:10 prior to storage at −20 °C or analysis.

### 2.4. Real-Time Polymerase Chain Reaction Analysis

Quantitative real-time polymerase chain reaction (qRT-PCR) analysis was performed in triplicate per gene and cDNA sample using 2.5 μL of diluted cDNA and 7.5 μL LightCycler 480 SYBR Green I master mix (Roche, Basel, Switzerland) and gene-specific forward and reverse primers at a final concentration of 0.625 μmol/L. The intron-spanning primer sequences are listed in Table 1. The plate-based real-time PCR amplification, detection and melting curve analysis of generated PCR products were performed using a LightCycler 480 Instrument II system (Roche, Basel, Switzerland). For PCR, 40 cycles of denaturation for 10 s at 95 °C, annealing for 15 s at 59 °C or 63 °C, followed by a thermal dissociation protocol for SYBR green detection was performed. For reaction validation and gene expression analysis the LightCycler 480 Instrument Operator’s Manual Software Version 1.5.1 was used (Roche, Basel, Switzerland). The sample mRNA expression normalization was done with an internal control gene index consisting of the geometric mean of the expression level of the housekeeping genes succinate dehydrogenase complex, subunit A, flavoprotein variant (*SDHA*), ribosomal protein L13a (*RPL13A*) and *β*2-microglobulin (*B2M*). The relative mRNA expressions of the target genes were determined by the comparative delta–delta C_T_ method considering the PCR efficiency of the gene of interest and the internal control genes [31].

### 2.5. Small Interfering RNA Transfection

For targeted gene silencing, small interfering RNA (siRNA) delivery into NHDF was performed by reverse transfection using the Lipofectamine 2000 transfection reagent (Thermo Fisher, San Diego, CA, USA). NHDF were maintained in antibiotic-free medium (2.9 × 10^5^ cells, 60 mm × 50 mm dish) and reverse transfected with the transfection mixture containing one of two separate silencer pre-designed siRNAs targeting Smad2, Smad3, Smad7 or a non-targeting, fluorescently labelled negative control siRNA diluted in Opti-MEM I Reduced Serum Medium (Thermo Fisher, San Diego, CA, USA) to yield a final siRNA concentration of 40–100 nmol/L per well. A total of 24 h post-transfection the siRNA transfection efficiency was monitored by fluorescence microscopy. A transfection efficiency of nearby 100% was considered for data analysis. When compared with negative control siRNA the siRNA that provided the most efficient inhibition per target gene determined via qRT-PCR was used for future experiments. For activin A treatment, transfected cells were serum-starved for 16 h and maintained in activin A-supplemented media for 6 h until cell lysis.

### 2.6. Radiochemical Xylosyltransferase Activity Assay

The determination of intracellular XT activity from cell lysates and extracellular enzyme activity from cell supernatants was performed by radiochemical determination of the enzyme-catalysed incorporation of UDP-[^14^C]-d-xylose (PerkinElmer, Foster City, CA, USA) into a silk fibroin acceptor protein as previously described [32]. The quantified disintegrations per minute (dpm) were referred to total DNA sample content and resemble the quantity of incorporated UDP-[^14^C]-d-xylose, which is proportional to the sample XT activity. For sample preparation of the extracellular XT activity the cell culture supernatant was collected, whereas for intracellular samples the same cell culture monolayer was lysed using 0.75 mL of a Nonidet P-40 (NP-40)-based buffer (50 mM TRIS, 150 mM NaCl, 1% NP-40, pH 7.8). After sample centrifugation (10,000 × *g*, 10 min at 4 °C), the supernatant was collected and stored at −20 °C until usage. The enzyme activities were measured 48 h and 120 h after cytokine treatment.

### 2.7. Statistical Analysis

The data are presented as mean values ± standard error of the mean (SEM). Statistical analysis of the variance between the experimental conditions was evaluated by non-parametric two-tailed Mann–Whitney U-tests using GraphPad Prism 7.0 (GraphPad Software, La Jolla, CA, USA) software. The lack of a Gaussian distribution was confirmed by checking normality visually (frequency distribution histogram) as well as by computing the Shapiro–Wilk normality test. The probability (P) values of less than 0.05 were considered as statistically significant. In the figures, the P values are indicated with asterisks and horizontal lines that connect the compared bars. Asterisks directly above the error bars of the treatment group indicate statistical differences between the treatment and control group, respectively.

## 3. Results

### 3.1. Identification of Pro-Fibrotic Mediators Regulating XT-I Expression in Fibroblasts

Previous work has demonstrated that TGF*β*1 is a potent inducer of the XT-I gene and protein expression in primary human dermal and cardiac fibroblasts [26,27]. We wanted to examine whether other pro-fibrotic cytokines and growth factors are able to modulate *XYLT1* and *XYLT2* mRNA expression of skin fibroblasts. Using a formerly established cell culture model [26], NHDF were incubated with recombinant human TGF*β*1 or different cytokines and growth factors (Figure S1). After 48 h of incubation we found significant increased activity and mRNA levels of myofibroblast marker XT-I (5.1 ± 0.9-fold and 4.2 ± 0.9-fold, both *p* < 0.0001; Figure S1A,B) and *ACTA2* mRNA level (2.4 ± 0.9, *p* < 0.0001; Figure S1C) in activin A-treated cells compared to the untreated controls, whereas no changes in *XYLT2* mRNA expression were observed (*p* = 0.06; Figure S1D). We were unable to demonstrate changes in XT activity or *XYLT1* mRNA expression after stimulation of NHDF with connective tissue growth factor (CTGF), angiotensin-II or endothelin-1 and could only show that some of them were able to exert regulatory effects on TGF*β*1-induced XT-I expression (Figure S1A,B). The treatment of NHDF with cytokines IL-4, IL-6 or IL-13 alone did not increase *XYLT1* mRNA expression either (Figure S2).

**Figure 1:**
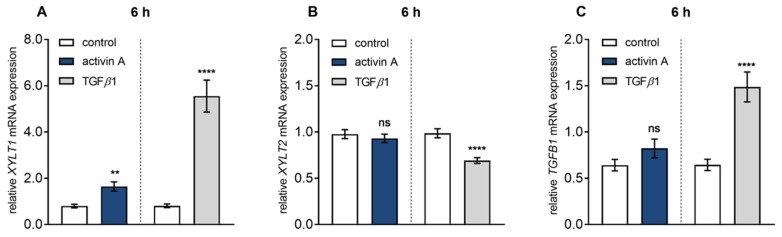
TGF*β* superfamily cytokine regulated *XYLT* isoform and *TGFB1* mRNA expression. Human primary fibroblasts (*n* = 3) were cultured the day before the experiment. Cells were serum-starved for 24 h and treated with activin A (10 μg/L; left side) or TGF*β*1 (10 μg/L; right side) for 6 h. The dashed line indicated that the activin A and TGF*β*1 experiments were performed independently. Relative *XYLT1* (**A**), *XYLT2* (**B**) or *TGFB1* (**C**) mRNA expression levels were analysed by quantitative real-time PCR. Shown values are means ± SEM for three biological and three technical replicates per experiment. Mann–Whitney *U* test: not significant (ns), *p* < 0.05 (*), *p* < 0.01 (**), *p* < 0.001 (***), *p* < 0.0001 (****).

In comparison to TGF*β*1 treatment, activin A-mediated *XYLT1* mRNA and XT activity regulation seemed to be less pronounced (7.7 ± 1.3-fold and 5.1 ± 0.9-fold vs. 4.2 ± 0.9-fold and 5.1 ± 0.9-fold, all *p* < 0.0001; Figure S1A,B). For direct comparison of the TGF*β*1 and activin A-mediated effects, serum-starved NHDF were treated with the same TGF*β*1 and activin A concentration of 10 μg/L and harvested after a short incubation time of 6 h, to minimalize the side effects due to secretion of secondary metabolites and mediators (Figure 1). Activin A treatment significantly increased *XYLT1* expression 2.1 ± 0.3-fold (*p* = 0.0018), whereas treatment with TGF*β*1 revealed a 6.9 ± 1.1-fold (*p* < 0.0001) increase of *XYLT1* expression (Figure 1A). In contrast, no stimulating effects (*p* = 0.46) could be observed for *XYLT2* mRNA expression after activin A or TGF*β*1 treatment of NHDF (Figure 1B). In order to investigate whether the induction of *TGFB1* transcripts may play a role in activin A-mediated *XYLT1* regulation, the *TGFB1* mRNA expression changes after TGF*β*1 and activin A treatment of NHDF were observed. While activin A treatment had no significant effect (*p* = 0.25) compared to the control, TGF*β*1 significantly auto-induced its mRNA expression 2.3 ± 0.3-fold (*p* < 0.0001) (Figure 1C). Taken together, these results show that activin A is a TGF*β*1-independent inducer of *XYLT1* mRNA expression and XT activity in NHDF.

### 3.2. Activin A Induces Transient XYLT1 mRNA Expression and XT Activity Increase via ACVRIB/ALK4

**Figure 2:**
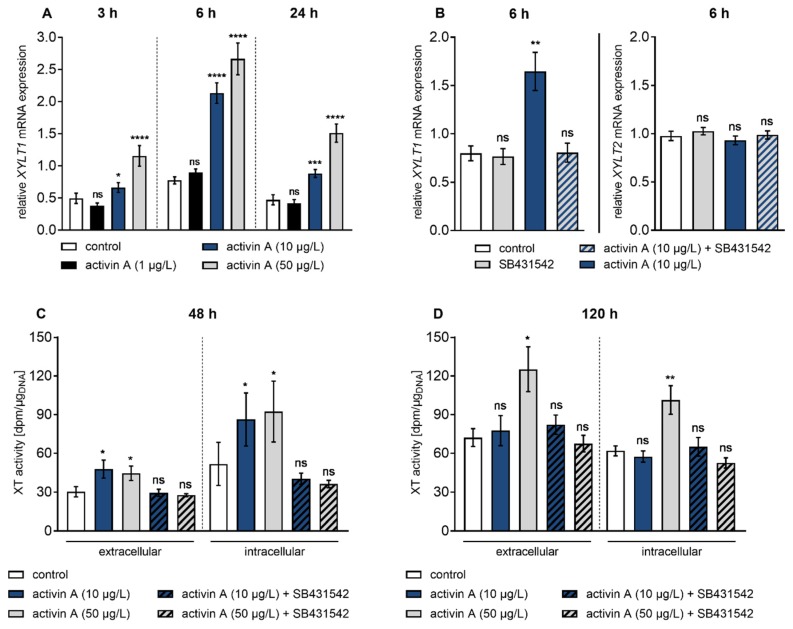
Time course of activin A-regulated *XYLT1* mRNA expression and XT activity. Human primary fibroblasts (*n* = 3) were cultured the day before the experiment. Cells were serum-starved for 24 h and treated with activin A or with activin A and the ALK4 inhibitor SB431542 (10 μmol/L) for the indicated time points. (**A**,**B**) Relative mRNA expression levels were analysed by quantitative real-time PCR. Shown values are means ± SEM for three biological and three technical replicates per donor-derived primary cell culture. Mann–Whitney *U* test: not significant (ns), *p* < 0.05 (*), *p* < 0.01 (**), *p* < 0.001 (***), *p* < 0.0001 (****). (**C**,**D**) XT activity was measured in cell supernatants and lysates by radiochemical enzyme assay and expressed as dpm per µg of DNA. Shown values are means ± SEM for three biological and one technical replicate per experiment. Mann–Whitney *U* test: not significant (ns), *p* < 0.05 (*), *p* < 0.01 (**).

Showing that activin A is a potent inducer of *XYLT1* expression under fibrotic conditions, we further examined the response of NHDF to various activin A concentrations in time-course experiments (Figure 2). The *XYLT1* mRNA expression levels were quantified 3 h, 6 h and 24 h post-treatment by qRT-PCR analysis (Figure 2A). The NHDF showed an early up-regulation of the *XYLT1* mRNA level at 3 h of activin A treatment at concentrations of 10 μg/L and 50 μg/L of 1.3 ± 0.3-fold (*p* = 0.01) and 2.3 ± 0.5-fold (*p* < 0.0001). This initial rise in *XYLT1* mRNA expression after 3 h was followed by a more pronounced expression increase in 10 μg/L and 50 μg/L activin A-treated cells of 2.7 ± 0.3-fold (*p* < 0.0001) and 3.4 ± 0.4-fold (*p* < 0.0001) after 6h in comparison to the untreated control fibroblasts. In comparison to control cells, activin A treatment at concentrations of 10 μg/L and 50 μg/L for 24 h increased the *XYLT1* expression level 1.9 ± 0.3-fold (*p* = 0.0002) and 3.2 ± 0.6-fold (*p* < 0.0001), respectively. The treatment with 1 μg/L activin A resulted in a 1.2 ± 0.1-fold (*p* = 0.01) increase in *XYLT1* mRNA expression after 6 h, though no expression changes were detectable after 3 h and 24 h treatment compared to the control (Figure 2A). These results demonstrate that activin A increases *XYLT1* mRNA expression in a time- and concentration-dependent manner in NHDF.

To confirm that the changes in *XYLT1* mRNA expression observed were caused by activin A treatment, cells were separately or simultaneously treated with the ALK4 inhibitor SB431542 [33,34] and activin A for 6 h (Figure 2B). The results showed that activin A induced a 2.1 ± 0.3-fold (*p* = 0.002) increase in *XYLT1* mRNA expression compared to the control that was completely abrogated by SB431542. Moreover, the single treatment of fibroblasts with SB431542 did not alter *XYLT1* basal expression (*p* = 0.79) compared to the vehicle-treated control cells. Treatment of NHDF with activin A or SB431542 did not alter *XYLT2* mRNA expression (Figure 2B). Together these results show an isoform-specific increase in *XYLT1* mRNA expression by activin A involving the ALK4 receptor.

Despite intracellular localization of XT-I, more than 90% of its enzyme activity is found in the culture supernatant, accumulating over time [25]. To examine if activin A-induced stimulation of *XYLT1* gene expression correlates with the changes in enzyme activity, extracellular and intracellular XT activity of NHDF were measured by radiochemical enzyme assay (Figure 2C,D). For this purpose, NHDF were treated with 10 μg/L and 50 μg/L activin A for 48 h and 120 h to promote detectable enzyme accumulation in the cell culture supernatant. In comparison to the untreated cells, the supplementation of activin A at different concentrations for 48 h increased the extracellular XT activity of NHDF 1.6 ± 0.3-fold (*p* = 0.03; 10 μg/L) and 1.5 ± 0.3-fold (*p* = 0.03; 50 μg/L), respectively. When analysing the XT activity from the corresponding cell lysates of former activin A-treated cells, a 1.7 ± 0.7-fold (*p* = 0.02; 10 μg/L) and 1.8 ± 0.7-fold (*p* = 0.04; 50 μg/L) enhancement in intracellular XT activity was detectable in comparison to the untreated controls (Figure 2C). Compared to the untreated control cells after 120 h, no inducible effects regarding extra- and intracellular XT activity were observed after incubation of NHDF with the activin A concentration of 10 μg/L. The activin A treatment of NHDF with a concentration of 50 μg/L increased the extracellular XT activity 1.7 ± 0.3-fold (*p* = 0.04), while the intracellular enzyme activity was significantly raised, 1.6 ± 0.2-fold (*p* = 0.001) (Figure 2D). In agreement with the gene expression data (Figure 2B), the usage of the ALK4 inhibitor SB431542 suppressed the activin A-mediated increase in extra- and intracellular XT activity (Figure 2C,D). These results reveal that the activin A-induced XYLT1 mRNA expression increase correlates time- and concentration-dependently with the changes in the extracellular and intracellular XT activity of NHDF.

### 3.3. ALK4–Activin A-Mediated XYLT1 mRNA Expression Requires MAPK Signalling Pathways

**Figure 3:**
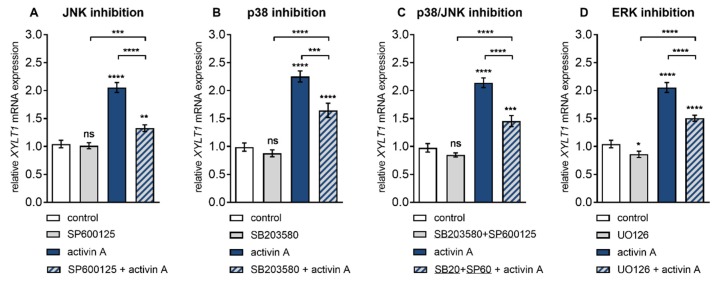
Inhibition of activin A-induced *XYLT1* mRNA expression by small-molecule inhibitors of MAPK pathways. Human primary fibroblasts (*n* = 3) were cultured the day before the experiment. Cells were serum-starved for 24 h and treated with or without activin A (10 µg/L) and (**A**) JNK inhibitor SP600125 (25 μmol/L), (**B**) p38 inhibitor SB203580 (10 μmol/L), (**C**) SP600125 (25 μmol/L) and SB203580 (10 μmol/L) or (**D**) ERK inhibitor UO126 (10 μmol/L) for 6 h. Relative mRNA expression level of *XYLT1* was analysed by qRT-PCR. Shown values are means ± SEM for three biological and three technical replicates per experiment. Mann–Whitney *U* test: not significant (ns), *p* < 0.05 (*), *p* < 0.01 (**), *p* < 0.001 (***), *p* < 0.0001 (****).

In order to understand the molecular mechanism of activin A–ALK4-mediated XT-I induction in NHDF, we investigated the underlying signalling pathways utilizing small-molecule inhibitors. The role of MAPK pathway on activin A-stimulated *XYLT1* expression was addressed by using SP600125, a broad-spectrum inhibitor of JNK [35]; SB203580, inhibitor of p38 MAPK [36]; and UO126, a highly selective ERK inhibitor [37] on NHDF in presence or absence of activin A (Figure 3).

We found that the activin A-mediated increases in *XYLT1* expression at 6 h post treatment could be diminished by JNK inhibitor SP600125 and p38 MAPK inhibitor SB203580 in a concentration-dependent manner (data not shown). Both, SP600125 and SB203580 significantly reduced activin A-induced *XYLT1* expression increase (2.3 ± 0.2-fold, *p* < 0.0001) 0.6 ± 0.04-fold (*p* < 0.0001) and 0.7 ± 0.1-fold (*p* = 0.0007), respectively (Figure 3A,B). In the presence of both particular inhibitors no regulatory effects regarding the basal expression of *XYLT1* (Figure 3A,B) and *XYLT2* (Figure S3A,B) were observed compared to the vehicle-treated control cells. Combinatory treatment of NHDF with JNK inhibitor SP600125 and p38 MAPK inhibitor SB203580 resulted in a significantly decreased activin A-mediated *XYLT1* mRNA level increase of 0.7 ± 0.05-fold (*p* < 0.0001) (Figure 3C). In the presence of both inhibitors no regulatory effects regarding the basal expression of *XYLT1* (Figure 3C) and *XYLT2* (Figure S3C) mRNA were observed compared to the vehicle-treated control cells.

In comparison to the vehicle and ERK inhibitor UO126 exclusively treated cells, the presence of activin A resulted in a particular 2 ± 0.1-fold (*p* < 0.0001) and 1.8 ± 0.1-fold (*p* < 0.0001) up-regulation of *XYLT1* mRNA expression, respectively (Figure 3D). Moreover, UO126 significantly decreased the basal *XYLT1* expression 0.8 ± 0.08-fold (*p* = 0.03) and the activin A-induced *XYLT1* expression increased (2 ± 0.1-fold, *p* < 0.0001) 0.7 ± 0.04-fold (*p* < 0.0001) of that of the respective controls (Figure 3D). In addition, we found a decreased basal mRNA expression of *XYLT2* of 0.9 ± 0.03-fold (*p* = 0.002) in UO126 inhibitor single-treated cells compared with those with the vehicle treatment (Figure S3D). These data show the involvement of MAPK JNK, p38 and ERK in ALK4–activin A-mediated *XYLT1* expression. Furthermore, the results indicate a role for MAPK ERK in the maintenance of basal *XYLT1* and *XYLT2* mRNA expression in NHDF.

### 3.4. Smad3 is Dispensable for Activin A-Induced XYLT1 mRNA Expression in NHDF

**Figure 4:**
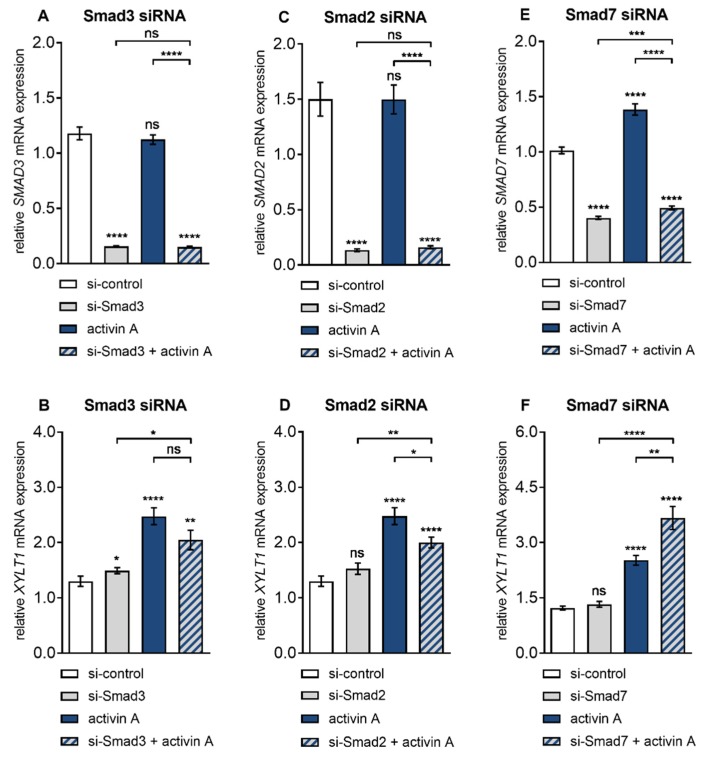
Basal and activin A-induced *XYLT1* mRNA expression were marginally affected by siRNA-mediated Smad knockdown. Human primary fibroblasts (*n* = 3) were cultured for 24 h before transfection with a negative control siRNA (si-control, 50 or 100 nmol/L) or siRNA targeting against (**A**,**B**) Smad3 (si-Smad3, 50 nmol/L), (**C,D**) Smad2 (si-Smad2, 50 nmol/L) or (**E**,**F**) Smad7 (si-Smad7, 100 nmol/L). A total of 24 h post-transfection, cells were serum-starved for 16 h and treated with or without activin A (10 µg/L) for 6 h. Relative *XYLT1* and *SMAD2*/*3*/*7* mRNA expression levels were analysed by qRT-PCR. Shown values are means ± SEM for three biological and three technical replicates per experiment. Mann–Whitney *U* test: not significant (ns), *p* < 0.05 (*), *p* < 0.01 (**), *p* < 0.001 (***), *p* < 0.0001 (****).

Earlier studies by Takagi et al. on NHDF showed an activin A-induced (10 μg/L) phosphorylation of Smad2/3 after 4 h cytokine treatment [10]. On the basis of these findings, we wanted to verify the impact of the canonical Smad pathway on basal *XYLT1* and *XYLT2* mRNA expression as well as on activin A-stimulated *XYLT1* expression increase by using siRNA knockdown experiments (Figure 4).

After Smad3 siRNA transfection, the basal *SMAD3* expression of the NHDF cells was knocked down 0.1 ± 0.01-fold (*p* < 0.0001), which did not change by additional activin A treatment compared to the negative control siRNA transfected cells (Figure 4A). Moreover, the Smad3 knockdown was selective, not affecting the expression levels of Smad2 or Smad7 (data not shown). Smad3 siRNA transfected NHDF showed an increased basal *XYLT1* expression of 1.1 ± 0.09-fold (*p* = 0.02) compared to the negative control siRNA transfected cells. Activin A treatment of control siRNA transfected NHDF resulted in a significant induction of *XYLT1* expression of 1.9 ± 0.2-fold (*p* < 0.0001). This induction did not significantly change (*p* = 0.08) in the presence of Smad3 knockdown. Therefore, Smad3 siRNA transfected NHDF treated with activin A exhibited an increased *XYLT1* expression of 1.6 ± 0.2-fold (*p* = 0.02) compared to the untreated control cells (Figure 4B). In contrast to basal *XYLT1* expression, the basal *XYLT2* expression level of Smad3 siRNA transfected cells fall below that of the respective siRNA controls (0.7 ± 0.02-fold, *p* < 0.0001) (Figure S4A). These results demonstrate that the induction of *XYLT1* expression by activin A is Smad3-independent, while basal *XYLT1* and *XYLT2* expression were reversely regulated by *SMAD3* expression.

After showing the dispensability of *SMAD3* in activin A-induced *XYLT1* expression we addressed the role of Smad2 in this context with the same experimental approach. NHDF transfected with Smad2 siRNA exhibited a highly significant *SMAD2* transcription decrease of 0.1 ± 0.01-fold (*p* < 0.0001) compared to negative control siRNA-treated cells, which did not change due to activin A supplementation (Figure 4C). Specific Smad2 siRNA knockdown in NHDF did not alter basal *XYLT1* expression (*p* = 0.08) compared to the negative control siRNA transfected cells, whereas the activin A-mediated *XYLT1* mRNA expression increase (1.9 ± 0.2-fold, *p* < 0.0001) was significantly diminished 0.8 ± 0.06-fold (*p* < 0.02) (Figure 4D). In comparison to the controls, the transfection of NHDF with Smad2 siRNA significantly up-regulated the basal *XYLT2* mRNA expression 1.2 ± 0.05-fold (*p* < 0.0001) (Figure S4B). These findings show that the induction of *XYLT1* expression by activin A is dependent on Smad2, whereas basal *XYLT1* expression is Smad2-independent. Furthermore, these results demonstrate a suppressive role of Smad2 on basal *XYLT2* expression in NHDF.

To confirm the results of differential and antagonistic regulation of basal and activin A regulated *XYLT1* and *XYLT2* mRNA expression by Smad2 and Smad3 we increased the cellular availability of activated Smads 2 and 3 by selective downregulation of Smad inhibitor Smad7. Activin A treatment for 6 h resulted in an increase in *SMAD7* expression of 1.4 ± 0.06-fold (*p* < 0.0001) compared to the untreated control cells. Compared to the negative control siRNA treatment of NHDF, the basal and activin A-induced *SMAD7* expression was decreased by Smad7 siRNA knockdown to a similar extend, 0.4 ± 0.01-fold (*p* < 0.0001) and 0.5 ± 0.02-fold (*p* < 0.0001), respectively (Figure 4E). Basal *XYLT1* expression was unaffected (*p* = 0.19) by Smad7 siRNA transfection compared to the negative control siRNA treated cells. The activin A treatment for 6 h significantly induced *XYLT1* expression 2 ± 0.1-fold (*p* < 0.0001) compared to untreated controls. This activin A-induced *XYLT1* expression increase was significantly enhanced 1.5 ± 0.1-fold (*p* < 0.004) after Smad7 deletion. Smad7 siRNA transfected NHDF treated with activin A exhibited an increased *XYLT1* expression of 2.8 ± 0.3-fold (*p* < 0.004) compared to the untreated control cells (Figure 4F). In contrast to the basal expression of *XYLT1*, basal *XYLT2* mRNA expression was induced 1.3 ± 0.05-fold (*p* < 0.0001) after Smad7 knockdown compared to the controls (Figure S4C). These results demonstrate that the induction of *XYLT1* expression by activin A can be enhanced by unavailability of Smad7. Additionally, the results show an inhibitory role of Smad7 on basal *XYLT2* expression in NHDF, while basal *XYLT1* expression is independent of *SMAD7* expression.

**Figure 5:**
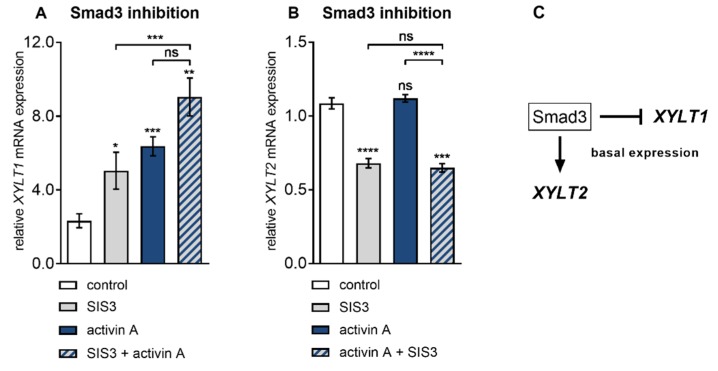
Differential regulation of basal *XYLT* isoform mRNA expression by Smad3. Human primary fibroblasts (*n* = 3) were cultured the day before the experiment. Cells were serum-starved for 24 h and treated with or without activin A (10 µg/L) in the presence or absence of the Smad3 inhibitor SIS3 (10 µmol/L) for 6 h. Relative (**A**) *XYLT1* and (**B**) *XYLT2* mRNA expression levels were analysed by qRT-PCR. Shown values are means ± SEM for three biological and three technical replicates per experiment. Mann–Whitney *U* test: not significant (ns), *p* < 0.05 (*), *p* < 0.01 (**), *p* < 0.001 (***), *p* < 0.0001 (****). (**C**) Schematic illustration of Smad3-dependent basal *XYLT2* and Smad3-independent basal *XYLT1* expression.

The finding that Smad3 inhibition by siRNA-mediated gene knockdown failed to properly impair activin A-induced *XYLT1* expression in NHDF was independently confirmed by pharmacologic Smad3 inhibitor SIS3 (Figure 5), which has been shown in previous works not to inhibit the Smad2 or MAPK pathways in human fibroblasts [38]. Compared to the vehicle-treated cells, the presence of SIS3 increased the basal *XYLT1* expression 2.1 ± 0.5-fold (*p* = 0.048) at 6 h post-treatment. NHDF treated with activin A for 6 h increased the *XYLT1* expression 2.6 ± 0.5-fold (*p* < 0.0001) of that from the untreated control cells (Figure 5A). In consistency with former results obtained from siRNA experiments (Figure 4B), the activin A-mediated expression increase in NHDF was not significantly impeded (*p* = 0.08) in the presence of the Smad3 inhibitor SIS3. In direct comparison to the SIS3 treatment alone, the activin A treatment up-regulated the *XYLT1* mRNA expression 1.8 ± 0.4-fold (*p* = 0.001) (Figure 5A). On the contrary, there was a significant decrease in the basal *XYLT2* expression of 0.6 ± 0.04-fold (*p* < 0.0001) in the SIS3-treated cells in comparison to the vehicle treated control cells (Figure 5B). These results confirm the former findings about Smad3 dispensability of activin A-mediated *XYLT1* mRNA expression increase and its suppressive role in basal *XYLT1* expression. In addition, we substantiated our findings of basal *XYLT2* mRNA expression dependency on *SMAD3* expression. Figure 5C shows a schematic diagram of these findings.

## 4. Discussion

The activity of GAG initiating key enzyme XT-I was found to be increased in the serum of patients with connective tissue diseases such as SSc or liver fibrosis [25,39]. Furthermore, long-term studies revealed that serum XT-I activity of SSc patients remains increased [25], corresponding to elevated PG metabolism and increased GAG content found in skin biopsies or cultured SSc fibroblasts [40,41]. Many TGF*β* superfamily ligands are potent mediators of ECM deposition and are highly up-regulated under fibrotic conditions [42]. Until now, studies have described an induction of *XYLT1* expression in fibrotic tissue or in cultured primary cells treated with TGF*β*1, IL-1*β* or thrombin [26,27,43,44,45,46,47], while the effect of other pro-fibrotic cytokines and their underlying signalling pathways remain unknown.

In the present study, we demonstrate for the first time that activin A, which was shown to be involved in wound healing processes and the pathogenesis of SSc [10,12], is a potent inducer of gene expression and activity of XT-I in dermal skin fibroblasts. Therefore, we provide evidence that one mechanism by which activin A contributes to dermal fibrosis in SSc is through the increase in *XYLT1* expression in NHDF, the major cells responsible for ECM remodelling [48].

We found that activin A-mediated *XYLT1* mRNA expression increase is time- and concentration-dependent, and considerably increased the intra- and extracellular XT enzyme activity in NHDF. The correlation of mRNA expression and enzyme activity increase regarding XT-I has been shown in this and numerous other studies using TGF*β*1-treated human dermal and cardiac fibroblasts [26,27,45,46,47]. Furthermore, we observed, in consistency with previous results in NHDF [25], that the extracellular enzyme activity of untreated cells increased over time due to the protein accumulation in the cell supernatant, while intracellular XT activity remains constant over the test period of 120 h. These findings support the hypothesis that two regulatory mechanisms exist to control enzyme activity in NHDF: One at the transcriptional stage and another operating post-translationally, shedding the Golgi-resident, constitutive active enzyme from the membrane for release to the extracellular space controlling the rate of PG biosynthesis by reducing the cellular enzyme amount [24,29]. In contrast to TGF*β*1, activin A treatment of NHDF results in a weaker *XYLT1* mRNA expression increase, which can be explained by the autocrine TGF*β1* signalling, which is responsible for sustaining or amplifying the fibrotic responses in the pathogenesis of SSc [7].

Former sequence analysis of the human *XYLT1* promoter region revealed several downstream transcription factors of the EGR-, SP1- or KLF-family to regulate *XYLT1* mRNA expression [28,29,43]. Giving the missing link between promoter analysis, identified transcription factors and upstream cellular signalling mediating *XYLT1* expression in response to TGF*β* cytokine stimulation of ALK receptors, we found a novel signalling axis involving MAPK and Smad proteins in activin A-mediated XT-I regulation in primary NHDF cells. Earlier studies by Takagi et al. on NHDF and SSc patients derived fibroblasts showed an activin A-induced phosphorylation of Smad2/3 after 4 h cytokine treatment, which could be reversed by ALK4 inhibition [10]. In agreement with the previous findings, we show here that the activin A-mediated effects on *XYLT1* mRNA and XT-I expression increases were ALK4-dependent in NHDF.

The ALK4–activin A-induced signal transduction can be promoted via canonical Smad or non-canonical, non-Smad pathways in human fibroblasts [10,16]. Regarding the role of non-canonical pathways, we found here for the first time that MAPK JNK, p38 and ERK are involved in ALK4–activin A-mediated *XYLT1* expression regulation in NHDF. The involvement of p38 MAPK in the TGF*β* pathway promoted XT-I mRNA and the activity increase were observed in cultured human cardiac fibroblasts stimulated with TGF*β*1 and corresponds with an elevated amount of digested GAG content in cardiac tissues from patients with myocardial fibrosis [27]. Additionally, in a recent study JNK and p38 were shown to be involved in growth factor thrombin or IL-1*β-*induced *XYLT1* mRNA expression increase in vascular smooth-muscle cells or primary chondrocyte cells [43,49].

While we demonstrated the contribution of the three MAPKs JNK, p38 and ERK in activin A-driven *XYLT1* expression increase in NHDF by the usage of small-molecule inhibitors, we were unable to detect a more pronounced decrease of activin A-mediated effects by simultaneous blocking of MAPK JNK and p38 compared to single p38, JNK or ERK inhibition. These data indicate reciprocal activation loops in the MAPK network itself or potential mechanisms of MAPK crosstalk and regulation by other signalling pathways [50,51]. The used SP600125 inhibitor concentration of 25 μmol/L in this study was previously shown not to block ERK1/2 and p38 phosphorylation [35], whereas blockade of p38 by SB203580 using an inhibitor concentration of 10 μmol/L resulted in a formerly identified increase on both ERK1/2 and JNK phosphorylation in primary hepatocytes and primary portal myofibroblasts [50]. Because of this cross-activation of MAPK signalling by MAPK inhibitors itself [35,50,52,53], it cannot be excluded that blocking of single MAPK JNK, p38 or ERK or simultaneous blocking of JNK and p38 MAPK pathways by the used small-molecule inhibitors in this study leads to a simultaneous activation of the other MAPKs driven by activin A. Therefore, no conclusion can be made on the basis of the observed relative extent of individual or combinatory MAPK inhibitions on the activin A-induced *XYLT1* mRNA expression increase.

Regarding the role of the canonical Smad signalling pathway, we suggest a Smad2-dependency of activin A-regulated *XYLT1* transcription, while Smad3 could be shown to be dispensable for *XYLT1* expression increase. Interestingly, we observed a slight inhibitory action of Smad3 with regard to the basal *XYLT1* expression of NHDF. In accordance with these findings, the full length *XYLT1* promoter does not provide a Smad binding element, except our group did identify the *XYLT1* promoter SNV with allele frequencies of 38%, detected in healthy blood donors, in which the base exchange c.-1088C>A results in a Smad3 transcription factor binding site that significantly reduced basal promoter activity by 48.1% ± 3% compared to promoter constructs without the mutation [54]. It might therefore be plausible that the slight increase in basal *XYLT1* expression observed in the Smad3-inhibited fibroblast cells of this study were due to this SNV. As former studies did not reveal any differences in serum enzyme activity in these blood donors harbouring the appropriate *XYLT1* promoter SNV c.-1088C>A [29], the corresponding *XYLT1* promoter SNV in this cells was therefore not considered further by sequencing for dividing into subgroups.

Additionally, the cell-type specific activation of individual MAPK JNK, p38 and ERK has been demonstrated to phosphorylate the regulatory linker region of Smad2 protein [13,49]. As Smad signalling is critical to fibroblast activation and fibrosis induced by activin A [49,55], inhibition of Smad2 signalling by usage of MAPK inhibitors might have contributed to the suppressed *XYLT1* expression increase in activin A-stimulated cells shown in this study. Several studies using human VSMC have demonstrated the dependency of PG synthesis on the phosphorylation of the Smad2 linker region. These studies especially highlighted Smad2 linker residue Thr220 regarding the transcriptional regulation of XT-I that can undergo phosphorylation by two of the three MAPKs p38, ERK and JNK after TGF*β* or thrombin stimulation of VSMC [49,56]. As our results show that activin A-mediated *XYLT1* expression increase is dependent on *SMAD2* expression and involves the activity of MAPK JNK, p38 and ERK, and thus possibly JNK, p38 and ERK initiate non-canonical phosphorylation of the Smad2 linker region, which could have an inducible effect on *XYLT1* mRNA expression in NHDF.

Deregulation of Smad7 expression has been associated with various human diseases, such as tissue fibrosis or systemic sclerosis [57]. Given the Smad3-dispensibilty of *XYLT1* expression regulation, we indirectly assumed the positive regulatory impact of increased Smad2 availability on *XYLT1* mRNA expression increase by depletion of Smad inhibitor Smad7. We could show that decreased *SMAD7* expression, mimicking SSc fibroblasts, increased activin A-driven *XYLT1* expression up-regulation but did not affect basal *XYLT1* expression. These data confirm the reliability of the used cell culture model and the role of XT-I as a marker for myofibroblast differentiation in fibrosis, as demonstrated in previous studies [26].

Former studies using activin A and a human neuroblastoma cell line demonstrated that the expression of some TGF*β* superfamily target genes induced by ALK4–activin A did not require promoter bindings of SMAD2/3 [10]. By taking into consideration that of the *XYLT1* promoter does not provide the according Smad binding site [29], but Smad2 linker phosphorylation was shown to enhance the nuclear localisation of Smad2 proteins [55], we presume that a potential activin A–ALK4 signalling pathway in NHDF is transduced by the MAPK JNK, p38 and ERK pathways and potential phosphorylation of the Smad2 linker region, promoting the nuclear entrance and favoured binding of Smad2 to the former identified transcription factors AP1 and SP1/3, known to increase *XYLT1* expression [28,29,58,59,60,61].

Interestingly, we found differential dependency of *XYLT* isoform expression on Smad3 and ERK. In comparison to basal *XYLT1* expression, which is increased by ERK and supressed by Smad3, basal *XYLT2* expression is increased by Smad3 and ERK in NHDF. This observation can be explained by considering the results of this current work and the previous *XYLT1* and *XYLT2* promoter analysis performed in our group. In contrast to the promoter region of *XYLT1*, the *XYLT2* promoter does not exhibit a binding site for the AP1 transcription factor, which plays a considerable role in the regulation of *XYLT1* gene by JNK and p38 [43], explaining why basal *XYLT2* expression was not influenced by the usage of p38 and JNK inhibitors in this study. While the *XYLT1* promoter exhibits numerous transcription-binding sites for EGR1, which was previously shown to response to non-canonical, Smad3-independent TGF*β* pathway MEK/ERK in SScF [60], the main transcription factors substantially involved in *XYLT2* promoter regulation are SP1/3 [29,30,62]. These findings are consistent with previously published data demonstrating dose-dependent inhibition of *SMAD3* promoter activity by ERK inhibitor UO126 that correlates with the inhibition of SP1/SP3 function by the same inhibitor [63]. Therefore, it is likely that apparent *SMAD3* expression due to ERK inhibition has led to diminish basal *XYLT2* expression. In accordance to Smad3 expression, which was shown to be down-regulated in former published data after TGF*β*1 treatment [64,65], *XYLT2* expression decreases in a *SMAD3*-dependent manner.

The knockdown of Smad2 in NHDF resulted in higher basal *XYLT2* expression, suggesting that Smad2 acts as a transcriptional suppressor. Additionally, the inhibition of *SMAD7* expression mimics the effect of Smad2 silencing on basal *XYLT2* expression in NHDF. It is described that active Smad proteins interfere with transcription factor complexes mediating repression of target gene expression [66]. Therefore, Smad2 and Smad3 binding to SP1 and their relative distribution to *XYLT2* gene expression regulation in NHDF require further clarification. With the knowledge that basal *XYLT2* expression relies on Smad3, the depletion of Smad2 in this study could have raised the endogenous ratio of Smad3 in comparison to Smad2. This could have led to the observed *XYLT2* expression increase in Smad2-depleted cells. The impact of siRNA-mediated knockdown of single Smad proteins on the endogenous Smad2/3 ratio and their respective complex formation has been shown in previous studies to change the outcome of target gene expression [67]. Future studies will be necessary to evaluate this reciprocal basal *XYLT2* expression regulation by Smads 2, 3 and 7 in NHDF.

## 5. Conclusions

Taken together, our data identify, for the first time, XT-I as a target of activin A-mediated pro-fibrotic effects in NHDF. *XYLT2* isoform expression was unchanged by activin A. We demonstrated the critical role for Smad2 and MAPK JNK, p38 and ERK in the underlying signalling pathways of increased *XYLT1* expression by activin A. Furthermore, we detected a differential regulation of basal *XYLT1* and *XYLT2* isoform transcription by MAPK and Smad proteins. This knowledge develops an enhanced understanding for their distinct expression pattern in tissues or under pathological conditions, and might be useful for isoform-specific activation or repression strategies of clinical relevance.

## Figures and Tables

**Table 1:** Primer sequences and annealing temperatures (T_A_) used for qRT-PCR analysis.

Gene	Primers	T_A_ (°C)	Product Size (bp)
*ACTA2*	5′-GACCGAATGCAGAAGGAG-3′ 5′-CGGTGGACAATGGAAGG-3′	59	169
*B2M*	5‘-TGTGCTCGCGCTACTCTCTCTT-3‘ 5′-CGGATGGATGAAACCCAGACA-3′	59	137
*RPL13A*	5′-CGGAAGGTGGTGGTCGTA-3′ 5′-CTCGGGAAGGGTTGGTGT-3′	63	165
*SDHA*	5′-AACTCGCTCTTGGACCTG-3′ 5′-GAGTCGCAGTTCCGATGT-3′	63	177
*SMAD2*	5′-ACAACAGGCCTTTACAGCTTCT-3′ 5′-GGAGGCAAAACTGGTGTCTCA-3′	63	239
*SMAD3*	5′-ACCATCCGCATGAGCTTC-3′ 5′-CACTGCAAAGGCCCATTC-3′	63	107
*SMAD7*	5′-AGATGCTGTGCCTTCCTC-3′ 5′-GTCTTCTCCTCCCAGTATGC-3′	63	135
*TGFB1*	5′-GCGATACCTCAGCAACC-3′ 5′-ACGCAGCAGTTCTTCTCC-3′	59	331
*XYLT1*	5′-GAAGCCGTGGTGAATCAG-3′ 5′-CGGTCAGCAAGGAAGTAG-3′	63	281
*XYLT2*	5′-ACACAGATGACCCGCTTGTGG-3′ 5′-TTGGTGACCCGCAGGTTGTTG-3′	63	139

## Supplementary Materials

The following are available online at https://www.mdpi.com/2218-273X/10/4/609/s1, Figure S1: Various Cytokine and growth factor treatments of NHDF. Figure S2: Interleukin treatments of NHDF. Figure S3: Inhibition of basal *XYLT2* mRNA expression by pharmacological inhibitors to MAPK JNK, p38 and ERK. Figure S4: siRNA-mediated Smads 2, 3 and 7 knockdowns.
