# Supplementary material for: Activin A-Mediated Regulation of XT-I in Human Skin Fibroblasts
Source: Biomolecules. 2020 Apr 14;10(4):609. doi: 10.3390/biom10040609 (PMC7226200; doi:10.3390/biom10040609)
Supplement: Supplementary file 1 [file biomolecules-10-00609-s001.pdf]

Supplementary Materials

Figure S1 (A-D)

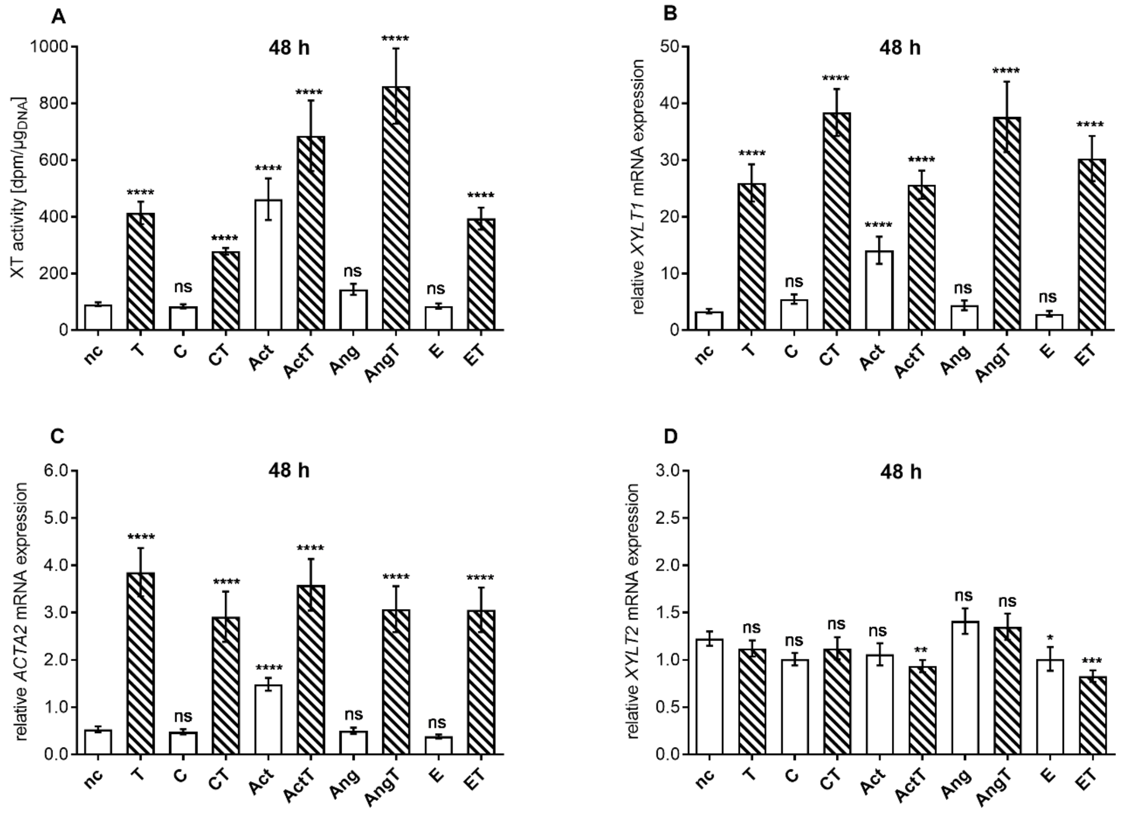

Figure S1. Various Cytokine and growth factor treatments of NHDF. Human primary fibroblasts (n = 4) were cultured the day before the experiment. Cells were serum-starved for 24 h and treated with vehicle (nc), activin A (Act; 50 µg/L), CTGF (C; 50 µg/L), angiotensin-II (Ang; 100 nmol/L), endothelin-1 (E; 100 nmol/L) alone or in combination with TGFβ1 (5 µg/L; T, CT, ActT, AngT, ET) for 48 h. (**A**) Extracellular XT activity was measured in cell supernatants by radiochemical enzyme assay and expressed as dpm per µg of total sample DNA. Relative (**B**) *XYLT1*, (**C**) *ACTA2* and (**D**) *XYLT2* mRNA expression levels were analysed by quantitative real-time PCR. Shown values are means ± SEM for three biological and three technical replicates per experiment. Mann-Whitney *U* test: not significant (ns), $P < 0.05$ (*), $P < 0.001$ (***), $P < 0.0001$ (****).

Figure S2 (A-C)

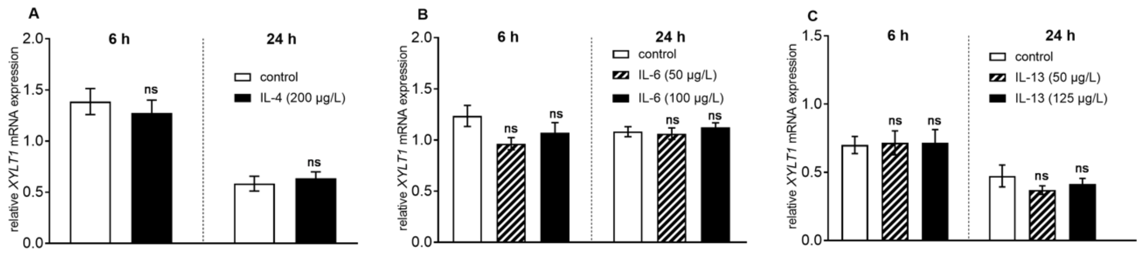

Figure S2. Interleukin treatments of NHDF. Human primary fibroblast cells were treated with (**A**) IL-4 (n = 3), (**B**) IL-6 (n = 2) or (**C**) IL-13 (n = 2) for 6 h and 24 h. Relative *XYLT1* mRNA expression levels

were analysed by quantitative real-time PCR. Shown values are means ± SEM for three biological and three technical replicates per experiment. Mann-Whitney *U* test: not significant (ns).

Figure S3 (A-D)

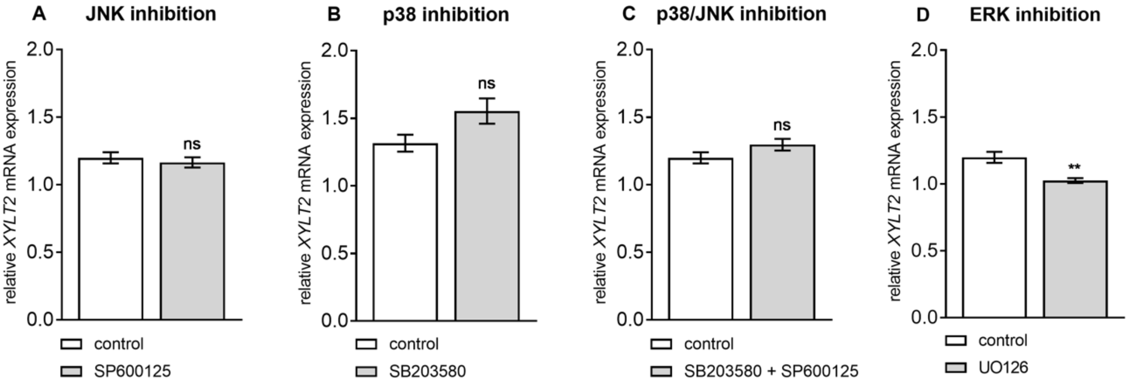

Figure S3. Inhibition of basal *XYLT2* mRNA expression by pharmacological inhibitors to MAPK JNK, p38 and ERK. Human primary fibroblast cells (n = 3) were cultured the day before the experiment. Cells were serum-starved for 24 h and treated with vehicle or (**A**) JNK inhibitor SP600125 (25 μmol/L), (**B**) p38 inhibitor SB203580 (10 μmol/L), (**C**) SP600125 (25 μmol/L) and SB203580 (10 μmol/L) or (**D**) ERK inhibitor UO126 (10 μmol/L) for 6 h. Relative mRNA expression level of *XYLT2* was analysed by qRT-PCR. Shown values are means ± SEM for three biological and three technical replicates per experiment. Mann-Whitney *U* test: not significant (ns), P < 0.01 (**).

Figure S4 (A-C)

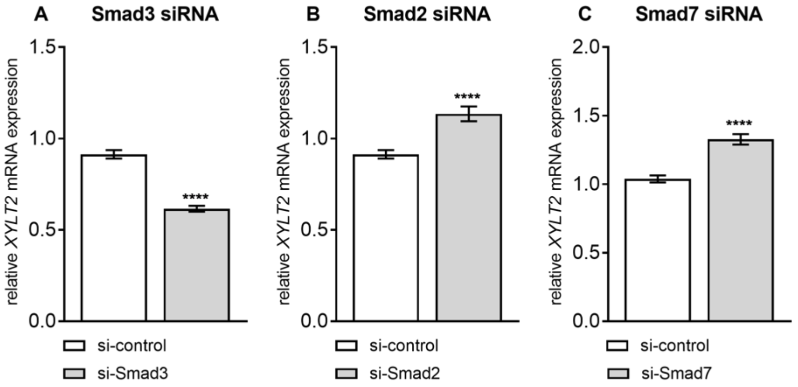

Figure S4. siRNA mediated Smads 2, 3 and 7 knockdowns. Human primary fibroblasts (n=3) were cultured for 24 h before transfection with a negative control siRNA (si-control, 50 or 100 nmol/L) or siRNA targeting against (**A**) Smad3 (si-Smad3, 50 nmol/L), (**B**) Smad2 (si-Smad2, 50 nmol/L) or (**C**) Smad7 (si-Smad7, 100 nmol/L). 24 h post-transfection cells were serum-starved for 16 h and maintained in serum-free media for additional 6 h. Relative *XYLT2* mRNA expression levels were analysed by qRT-PCR. Shown values are means ± SEM for three biological and three technical replicates per experiment. Mann-Whitney *U* test: P < 0.0001 (****).
